# Exploring the influence of the nanoporous structure of nickel-based superalloy membranes on emulsification performance

**DOI:** 10.1016/j.ijpx.2025.100369

**Published:** 2025-07-27

**Authors:** Daniel Jupke, Janik Marius Lück, Joachim Rösler, Jan Henrik Finke, Arno Kwade

**Affiliations:** a Institute for Particle Technology (iPAT), Technische Universität Braunschweig, Volkmaroder Straße 5, 38104 Braunschweig, Germany; b Center of Pharmaceutical Engineering (PVZ), Technische Universität Braunschweig, Franz-Liszt-Straße 35A, 38106 Braunschweig, Germany; c Institute for Materials Science (IfW), Technische Universität Braunschweig, Langer Kamp 8, 38106 Braunschweig, Germany

**Keywords:** Ni-based superalloy, Metal membrane, Premix membrane emulsification, Multi-stage emulsification, Membrane structure, Nanoemulsion, High pressure

## Abstract

Nanoporous structures made from nickel-based superalloy are fairly new and not thoroughly studied membrane materials for premix membrane emulsification. They show a different kind of pore structure than other membranes typically used in this process with a network of elongated, interconnected pores (150–400 nm). Two different membrane structures, resulting from different creep strains, times and temperatures during production, were investigated for their performance in premix membrane emulsification. The membranes were used in a system with a fixed process pressure, varying specific energy input via pressure or number of emulsification cycles. Furthermore, membranes with different manufacturing parameters and thicknesses were used. Both membrane structures achieved monomodal droplet size distributions with median droplet sizes under 500 nm in one emulsification cycle. The results indicate that while all droplet sizes fall within a comparable range, the pore sizes still play a significant role, with finer pores resulting in smaller droplets but broader droplet size distribution that showed minimal further breakup after repeated passes. The larger, more irregular pores showed the ability to further breakup droplets with increasing emulsification cycles, broadening their distribution. The findings also suggest that a pressure increase activates smaller pores that seem to remain inactive for emulsification at lower pressures, facilitating more transport and droplets breakup. Results underscore the critical role of elongational flow at the membrane inlet in promoting droplet breakup. This study strengthens the theory that droplet breakup in premix membrane emulsification requires droplets to be stretched as they enter the membrane, then breakup either spontaneously by surface instabilities when remaining in this elongated state for a sufficient time or deterministically by mechanical stresses such as shear caused by a tortuous channel.

## Graphical abstract

**Figure:**
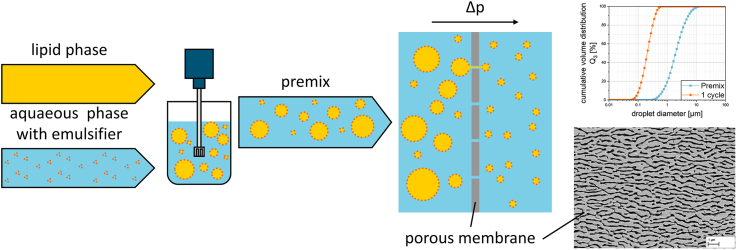

## List of symbols

A: active membrane area [m^2^]
cce: cylindrical capillary equivalent pore diameter [m]
$d_{h}$: hydraulic diameter [m]
$E_{v}$: specific energy [J/m^3^]
h: pore height [m]
n: number of emulsification cycles [−]
Re: Reynolds number [−]
s: membrane thickness [m]
v: flow speed [m/s]
$\dot{V}$: flow rate [m^3^/s]
w: pore width [m]
$X_{y}$: droplet diameter corresponding to y vol% of a cumulative volume distribution [m]
Δp: pressure drop across membrane [bar]
ρ: density of the emulsion [kg/m^3^]
ε: membrane porosity [−]
η: dynamic viscosity of the premix [mPa s]
λRe: Darcy friction factor [−]

## 1 Introduction

**Fig. 1:**
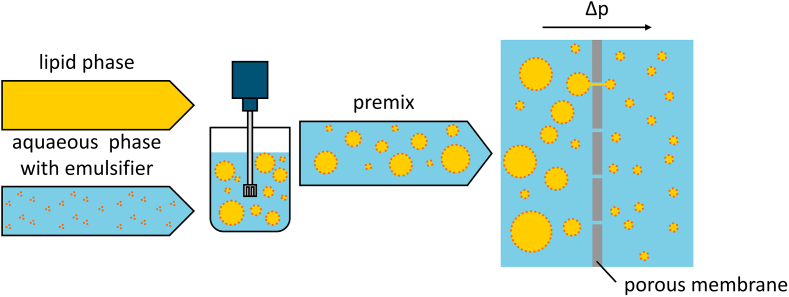
Schematic flow diagram of the premix membrane emulsification process.

Emulsions play an import role in foods, cosmetics and pharmaceutics. In pharmaceutical research, poorly water-soluble active ingredients are a challenge in the development of modern dosage forms. Possible dosage forms for such lipophilic drugs are parenteral oil in water O/W-nanoemulsions. For this form of application, a small droplet size of typically around 300 nm and a narrow droplet size distribution in the range of 200–450 nm should be provided (Driscoll, 2002) with less than 0.4 % of the total lipid content forming droplets over 5 μm (Driscoll et al., 1995). One possible process to achieve these target parameters is premix membrane emulsification, for which a coarse premix emulsion is pressed through a membrane (see Fig. 1). This process was first described by Suzuki et al. in 1998 (Suzuki et al., 1998) and has been the topic of ongoing research in the following years. The result of the emulsification strongly depends on the membrane, process parameters and formulation used. If the parameters mentioned are well selected, nanoemulsions suitable for intravenous application can be produced.

The membrane is of crucial importance in this process. Typical membranes for premix membrane emulsification are glass- ceramic- or polymer-based (Nazir and Vladisavljević, 2021) and limiting in terms of the formulations and process conditions, especially the pressure that can be applied. One of the most widely studied membrane materials is shirasu porous glass (SPG) (Nakashima et al., 1992) with a branched, interconnected pore structure. These type of membranes are reusable but expensive and quite fragile (Gehrmann and Bunjes, 2018). Ceramic membranes have been the subject of fewer studies than glass membranes, but it has already been demonstrated that membrane emulsification can be successfully carried out with this material, which has high chemical and thermal resistance (Gehrmann and Bunjes, 2018; Jing et al., 2005; Nishihora et al., 2020). Since the group of polymer membranes comprises many different materials, the structures of these membranes vary greatly depending on the material and manufacturing process from cylindrical pores of straight-through membranes made using track etching to more complex interconnected pore structures produced by phase inversion (Tan and Rodrigue, 2019a, Tan and Rodrigue, 2019b). Polymer membranes are usually disposable (Nazir and Vladisavljević, 2021). The use of metal membranes, on the other hand, enables the application of significantly higher process pressures while still offering the possibility of reusability after appropriate cleaning. Previous work has shown the applicability of nickel sieves for premix membrane emulsification (Nazir et al., 2011). Still, the achievable droplet size was limited due to the size of the pores, which could be manufactured at this time. Nevertheless, the use of metal membranes offers the possibility of extending the pressure range from the low pressure range, which is usually limited by strength and structural integrity of the membranes used in premix membrane emulsification, to higher pressures. This change offers the opportunity to increase the mass flow significantly, as higher pressure usually results in higher throughput. It also makes it possible to achieve different flow regimes and potentially gain new insights into droplet breakup.

Droplet breakup mechanisms are subject of ongoing research and are strongly depending on the emulsification system. In a microfluidic device, droplet breakup in a porous glass membrane structure could be shown to be due to local shear forces, splitting due to interfacial tension effects, and due to displacement and constriction of neighboring droplets (van der Zwan et al., 2006). As stated before for the premix membrane emulsification the membrane is of fundamental significance. Both pore size and structure play a decisive role in the achievable droplet size. Smaller pores lead to smaller droplets (Umeda et al., 2025; Zhou et al., 2009). For Nickel sieves it has already been shown, that the breakup in straight-through pores is less affected by shear than is the case with interconnected pore systems (Nazir et al., 2013). This stems from the fact that interconnected pores usually do not provide a direct path through the membrane, as straight-through pores do, but expose the droplets to more shear stress due to changes in direction instead. The process of droplet breakup due to change in direction was already shown for T-junctions and obstacles blocking the way of droplets in an otherwise ideal channel (Link et al., 2004; Protière et al., 2010). Navarro et al. used a moving-frame boundary-integral algorithm to show that pore networks with bifurcation can cause more effective droplet breakup than pores with only constrictions or pores without either (Navarro et al., 2021).

In premix membrane emulsification, the process of pressing the emulsion through the membrane multiple times is called repeated or multi-stage premix emulsification (Nazir et al., 2010). The rational for this is further droplet breakup with additional cycles. Under the same pressure conditions without fouling, an increase in mass flow can be expected, as less of the energy input is used for droplet breakup (Vladisavljević et al., 2004).

A nanoporous membrane based on the single-crystalline nickel-based superalloy CMSX-4 is a promising development in the field of metal membrane production. The alloy, which originally comes from the field of turbine blade production, consists of a two-phase structure in the heat-treated initial state, which is set by solution annealing and subsequent precipitation heat treatment (Rösler et al., 2005). Approximately 225 nm sized, cube-shaped γ’-precipitates are embedded in a γ-matrix (Lück and Rösler, 2022). In the following thermomechanical treatment, also imposed on the material when used as a turbine blade, a lamellar structure can be produced at an elevated temperature and stress with the help of the so-called rafting process. This microstructural transformation is based on the fact that, under load in the [001]-direction, there is a more energetically favorable state for the precipitation microstructure, so that the horizontal γ-matrix channels are enlarged at the expense of the vertical (Kamaraj, 2003; Kamaraj et al., 2001; Kohnke et al., 2018; Matan et al., 1999; Rösler and Voelter, 2018; Touratier et al., 2009). Depending on the creep duration and creep strain, a bicontinuous network develops in this raft formation process, in which the γ’-precipitation phase is interconnected in all three dimensions. Important factors influencing the rafting process are the temperature, stress, load direction and lattice mismatch of the alloy. In the process of membrane fabrication, the resulting microstructure of the γ-matrix is of enormous importance, as it is dissolved in the subsequent extraction process, resulting in an open-porous structure from the remaining γ’-phase (Kamaraj et al., 2001; Rösler et al., 2005; Rösler and Mukherji, 2003; Rösler and Voelter, 2018).

In an initial study by Kohnke et al. (Kohnke et al., 2018) on the use of nanoporous nickel membranes for premix membrane emulsification, membranes had a pore size of 250–400 nm and emulsion was carried out flow rate-controlled. After one emulsification cycle, droplet sizes between 434 nm (0.5 mL/s) and 324 nm (1.4 mL/s) were achieved. Furthermore, the nanoporous nickel membranes were compared with, among others, 0.01–0.02 mm thick polyester (PE) membranes with a nominal pore size of 200 nm. Although the PE membranes were able to achieve a smaller droplet size of 413 nm (0.5 mL/s) to 379 nm (1.4 mL/s) after the first cycle, they showed fouling behavior after 21 cycles, which could not be observed using the nickel membranes.

This work features an optimized Ni-based superalloy membrane with a reduction in pore size to approx. 120–150 nm. This was achieved by an optimized heat treatment route (Lück and Rösler, 2022), encompassing a temperature reduction from 1000 to 950 °C and stress increase from 170 MPa to 250 MPa (Lück and Rösler, 2023). By an intensive emulsification study, the hypothesis is tested, whether the optimization of the pore channel width leads to an improvement in emulsification performance. Moreover due to the novelty of the membranes, systematic research regarding the strengths and limitations of the membranes with respect to membrane emulsification is missing, especially also towards the understanding of droplet breakup mechanisms and industrial application. Accordingly, this study focuses on the influence of pore structure, emulsion formulation and process parameters during emulsification. The membrane thickness and pore geometry are systematically studied towards their effect on droplet size distribution.

## 2 Materials and methods

### 2.1 Manufacturing of Ni-based superalloy membranes

The single-crystalline nickel-based superalloy CMSX-4 was used to fabricate nanoporous membranes. For this purpose, a creep sample was machined from a rod with a length of 220 mm and a diameter of 21 mm. The material was produced by the manufacturer Access Aachen e.V. using the Bridgeman process (sample ID starting with “*Z*-”). The deviation of the [001]-orientation from the rod centerline was determined by the Eigenmann X-ray laboratory using Laue diffraction and is less than 11° for the rods used. One of the four creep samples used was made from a single-crystal plate (Lück and Rösler, 2025). The deviation of the [001]-orientation from the sample centerline was measured using electron backscatter diffraction (EBSD) in a scanning electron microscope (SEM) (FEI Helios NanoLab 650) and is less than 20° (sample ID starting with “A-”).

All samples starting with “Z-“ were first solution annealed and precipitation heat treated at the following temperatures and durations: 1277 °C / 2 h + 1288 °C / 3 h + 1296 °C / 3 h + 1304 °C / 2 h + 1313 °C / 2 h + 1316 °C / 2 h + 1318 °C / 2 h + 1321 °C / 2 h + AC to RT + 1140 °C / 30 min + AC to RT (AC: air cooling; RT: room temperature). The γ’-precipitate size optimized for membrane production after the heat treatment is approx. 224 ± 52 nm, the channel width between the γ’-precipitates is approx. 35 ± 19 nm (Lück and Rösler, 2025, 2022). Samples starting with “A-“ were solution annealed using 1277 °C / 2 h + 1288 °C / 3 h + 1296 °C / 3 h + 1304 °C / 2 h + 1313 °C / 2 h + 1316 °C / 2 h + 1318 °C / 2 h + 1321 °C / 2 h + AC to RT followed by a precipitation heat treatment 1080 °C / 4 h + AC to RT. Following the heat treatment, creep rupture specimens with a rectangular cross-section and a width of 16 mm were produced. The thickness of the specimens varied between 6.3 and 7 mm, the total length between 120 and 138 mm. An M16 x 2 thread was used for mounting. The creep specimens were then subjected to a creep test at constant temperature and stress in accordance with Table 1. Subsequently, sheets with a thickness of 0.45 mm were eroded from the middle area of the creep test specimen using wire erosion. Finally, discs with a diameter of approx. 15.5 mm were eroded from these sheets. To prepare the membrane samples, the sheets were ground down to the desired thickness (including P2500) and electrochemically etched to dissolve the γ-phase, leaving a channel-like, porous structure of γ’-phase as a membrane. A solution consisting of 800 mL H_2_O, 8 g (NH_4_)_2_SO_3_ and 8 g C_6_H_8_O_7_ was used in combination with a voltage of 1.3 V for the dissolution. A detailed description of the experimental setup can be found in (Lück and Rösler, 2025; Rösler et al., 2005).

**Table 1:** Loading conditions of the creep test samples and mean pore channel width and length after the dissolution of the γ-phase.

Specimen-ID	Temperature [°C]	Creep strain [MPa]	Time [h]	Elongation [%]	Pore width [nm]	Pore length [nm]
Z1	950	250	212	1.3	143 ± 105	356 ± 687
Z2	950	250	282	2.7	144 ± 114	363 ± 615
Z3	950	250	398	3.9	137 ± 94	426 ± 775
A4	1000	170	839	4.7	342 ± 142	606 ± 514

In order to determine the average channel width and length in the extracted state, 6 images were taken at a magnification of 8 k using a scanning electron microscope (ZEISS LEO 1550 GEMINI) in dendritic and interdendritic areas. The images were then cropped to a size of 688 × 688 pixels, binarized using the Fiji (Schindelin et al., 2012) plugin Trainable Weka Segmentation (Arganda-Carreras et al., 2017) and finally measured using the line intersection method. A detailed description can be found in (Lück and Rösler, 2025, 2023) and [20].

**Fig. 2:**
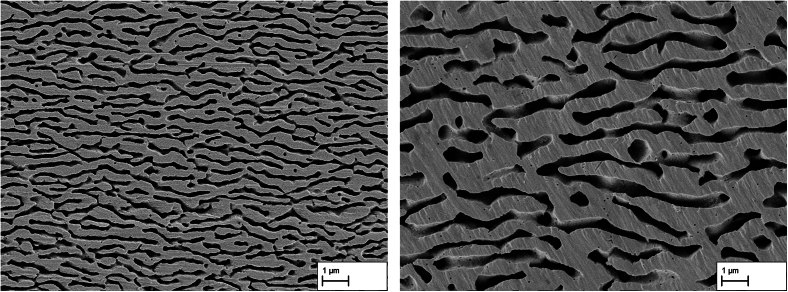
Membrane pore structure of the sample Z1 after selective phase dissolution of the γ-phase. The pore structure shows elongated pores with a channel width of approx. 143 ± 105 nm (left), and pore structure of the sample A4 with shorter and increasingly round pores and a channel width of approx. 342 ± 142 nm (right).

The extracted membranes can essentially be divided into two types. *Z*-membranes that were thermomechanically treated in the creep test at 950 °C and 250 MPa have an elongated pore structure, in which the pore length is a multiple of the pore width with an aspect ratio of the pores of around 2.5, giving the impression of the pattern of zebra stripes (Fig. 2, left). In contrast, A-membranes being thermomechanically treated at 1000 °C and 170 MPa have increasingly shorter and rounder pores with an aspect ratio of the pores of around 1.8, conveying the impression of the cross-section of an ant hill (Fig. 2, right). For ease of understanding, the membranes have been named after the natural structures they resemble (Z for zebra stripes and A for anthill cross-section). Please note that samples crept at 950 °C and 250 MPa originate from a study by Lück et al. where the mechanical strength of the nanoporous membranes depending of the creep strain was investigated (Lück and Rösler, 2025).

### 2.2 Premix membrane emulsification

#### 2.2.1 Premix formulation

An O/W emulsion was used for emulsification. The dispersed phase consisted of 10 %(*w*/w) medium chain triglycerides (Migylol® 812, Sasol, Hamburg, Germany) stabilized by 7.5 %(w/w) sodium dodecyl sulfate (SDS Pellets, Carl Roth, Karlsruhe, Germany) in ultrapure water as the continuous phase (experiments in section 3.1–3.7). Additionally, emulsions stabilized with only 0.75 %(w/w) SDS were studied in subchapter 3.7. The premix was produced in 1 L batches for each experiment with a T 25 digital ULTRA-TURRAX using an S 25 N - 25 G dispersing tool (both IKA, Staufen, Germany) at 10,000 rpm (circumferential speed 8.9 m/s) for 1 min.

#### 2.2.2 Emulsification setup

**Fig. 3:**
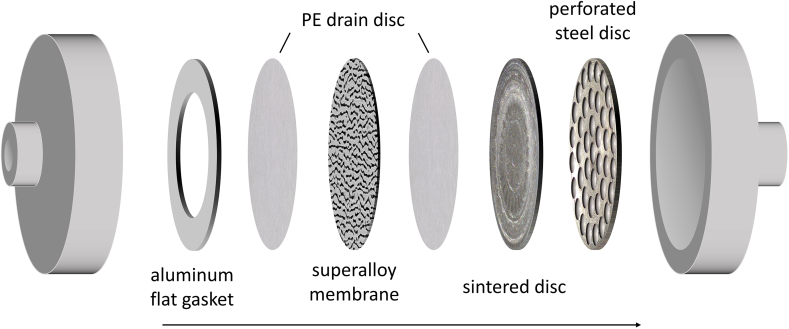
Schematic diagram of the sequence of flat gasket, membrane, support discs and drain discs in the membrane holder.

Emulsification was performed with a custom-built premix membrane emulsification setup. The process was carried out with a constant pressure supplied by using a pressure intensifier (Ausgleichszelle, SITEC-Sieber Engineering, Maur, Switzerland). The membrane was held in place by a custom stainless steel membrane holder with a diameter of 19 mm. To stabilize the membrane in case of higher pressure, two porous support discs made from stainless steel supported the Ni-based superalloy membrane. The first one was a sintered disc made from pressure formed 316 stainless steel powder. The production process resulted in a structure with pores around 2 μm in size (Gehrmann and Bunjes, 2016). The second was a perforated stainless steel disc with straight through holes of 3 mm. Both support discs were manufactured to fit the membrane holder with little tolerance in the recess. Before and after the superalloy membrane, a polyethylene drain disc (Cytiva, Marlborough, United States of America) was placed for ease of separation of the superalloy membrane and the sintered disc and to keep coarse particulate from contaminating and blocking the membrane. In front of the membrane, an aluminum flat gasket was placed to ensure a constant inflow area of the superalloy membrane. The sequence of flat gasket, membrane, support discs and drain discs in the membrane holder is shown in Fig. 3 The setup allowed a digital measurement of the applied pressure and the resulting mass flow of the product emulsion. Before and after emulsification, the setup was flushed with water and ethanol, to remove residue of oil or emulsifier in the system. The membrane was sonicated in an ultrasonic bath filled with ethanol for 10 min after the experiment.

#### 2.2.3 Droplet size analysis

The droplet size distribution was analyzed via laser diffraction spectroscopy using a Mastersizer 3000 equipped with the automated dispersion unit Hydro MV (Malvern Panalytical, United Kingdom). Samples were diluted in deionized water to a laser obscuration between 1 and 10 %. For the calculation of the volume distribution, the Mie theory (Mie, 1908) was used with 1.46 as the refractive index for the dispersed oil droplets and 1.33 for the continuous aqueous phase. The width of the droplet size distribution was evaluated using the span (Eq. 1).

(1) $$\mathrm{span}=\frac{X_{90}-X_{10}}{X_{50}}$$

#### 2.2.4 Viscosity measurement

The viscosity was measured using a rotary rheometer (MCR 302, Anton Paar GmbH, Graz, Austria) equipped with a rotating coaxial cylinder inside a cup forming a Searle system (CC27, Anton Paar GmbH, Graz, Austria). The tests were run at 25 °C and shear rates from 0.1 to 1000 1/s.

#### 2.2.5 Emulsification using varying support discs

In order to show that the main droplet breakup was performed by the novel Ni-based superalloy membranes, experiments were performed with varying usage of the support discs. Therefore, the emulsification setup was equipped with varying membrane and disc configurations. In addition to the normal setup described in 2.2.2, emulsification was once performed with only the perforated stainless steel disc; once with the sintered disc and the perforated steel disc and once with the superalloy membrane together with the perforated steel disc.

#### 2.2.6 Investigation of membrane structure and thickness

The two different structures described in 2.1 were used for emulsification at a constant pressure of 120 bar as multi-stage emulsification, performing five consecutive cycles. The pressure was chosen based on the maximum pressure that was achievable by the emulsification setup used in previous works (Kohnke et al., 2018).

Additionally using the two different membrane structures, one emulsification cycle was performed at process pressures from 50 to 250 bar. The experiments were performed consecutively in order of increasing pressure. A further experiment was carried out with a second Z1 membrane, in which five consecutive cycles were performed at 100, 200 and 300 bar. The aim of this was to better assess the influence of fouling on the structure, as the membrane was cleaned between the pressure changes. This was not carried out in the experiment with the increasing pressures.

Both membrane structures were examined for the influence of membrane thickness. For this the three different thicknesses 0.3; 0.2 and 0.1 mm were produced. The thickness of the membranes was adjusted by grinding the membranes to a specific value. For clarification, a list of the experiments carried out is shown in Table 2.

**Table 2:** Process parameters of emulsifications using metal membranes.

Pressure [bar]	Membrane ID	Number of cycles	Membrane thickness [mm]
120	A4	5	0.1–0.3
120	Z1	5	0.22
120	Z2	5	0.1–0.3
120	Z3	5	0.22
120	PE	5	0.014
50–250	A4	1	0.3
50–250	Z1	1	0.22
100; 200; 300	Z1	5	0.22

#### 2.2.7 Emulsification using a polymer membrane

In order to better compare the results of the superalloy membranes with a common polymer membrane of similar pore size, a track etched polyester (PE) membrane (Pieper Filter, Bad Zwischenahn, Germany) with 200 nm pores was used. The emulsification was performed at 120 bar for 5 consecutive cycles (see Table 2).

#### 2.2.8 Calculations

In this work, different calculations were used to characterize or quantify parameters relevant for the emulsification. The specific energy ($E_{v}$) input was calculated according to Eq. (2) with Δp being the pressure drop across the membrane, i indicating the use of different pressures and n the number of cycles.

(2) $$E_{v}=\sum_{i=1}^{n}\Delta p_{i}$$

It is important to note, that the specific energy input in case relates to the total amount of energy transferred into the system and not the maximum energy that a droplet experiences during breakup. In this way, multiple stress events with little energy can result in the same specific energy input as one stress event with high energy.

A statement about the flow conditions in a given system can be made using the Reynolds number (Re) given in Eq. (3). It is a dimensionless number calculated by the ratio of inertia forces to viscous forces. The dimensionless number is calculated using the density of the emulsion ρ, the flow speed v and the dynamic viscosity of the premix η. For the characteristic length, the hydraulic diameter $d_{h}$ (4) calculated from a mean of the heights h and widths w of the pores was used.

(3) Re=ρ·v·dhη

(4) $$d_{h}=\frac{2\cdot h}{1+\frac{h}{w}}$$

For flow in a pipe, typically a Recrit value of 2300 is used for indicating the transition from laminar to turbulent flow (Rotta, 1956). However, this is not a hard limit as laminar conditions can be observed for Re over and the beginning of turbulent flow for Re under Recrit.

The Darcy–Weisbach equation can be used for a simplified estimation of the pressure loss to be expected when flowing through a packing such as filtration or membrane emulsification. In this approach, the membrane is considered as a packing of parallel, unconnected capillaries with the same mean velocity. In Eq. (5) λRe represents the Darcy friction factor and s represents the thickness of the membrane.

(5) Δp=λRe·ρ2·v2·sdh

The Darcy friction factor, which provides information about the pressure drop of a flow due to flow resistance in a straight pipe, can be estimated under laminar conditions using Eq. (6).

(6) λRe=64Re

For quantitative comparison of pores sizes in membranes, Hinze formulated Eq. (7) to calculate the cylindrical capillary equivalent pore diameter (cce) based on the Hagen-Poiseuille equation, Darcy's law and the flow resistance of membranes related to their length using the flow rate $\dot{V}$, the active membrane area A and the membrane porosity ε.

(7) $$\mathrm{cce}=\sqrt{\frac{32\cdot s\cdot \dot{V}\cdot \eta }{A\cdot \Delta p\cdot \varepsilon }}$$

The cce provides a value for the pore diameter, if the membrane were composed of only straight tubes running form one end to the other without any tortuosity.

## 3 Results and discussion

### 3.1 Influence of support discs

**Fig. 4:**
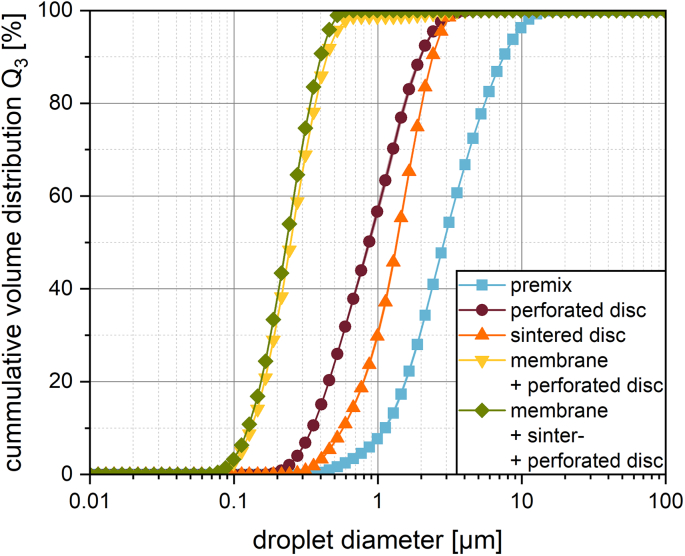
Droplet size distribution achieved using different parts of the custom premix membrane emulsification setup at 120 bar.

The results for the influence of the support discs show, that the droplet breakup was less intense when using only the perforated steel disc or when using the perforated steel disc together with the sintered steel disc. Nevertheless, there was some droplet breakup detected (see Fig. 4). The median size of 1.36 μm for the droplets produced corresponds with the reported pore size in the sintered disc of around 2 μm. When using no sintered disc, the droplet size was even lower. This cannot be explained via the pore sizes alone. The smaller droplet size could potentially have been achieved due to the higher speeds and the turbulence reached in this setup similar to high pressure orifice emulsification. The mass flow of the emulsion increased from 0.111 ± 0,086 g/s using the sintered disc together with the perforated disc up to 1.580 ± 0,632 g/s when using the perforated disc alone. This change in flow rate by a factor of almost 15 corresponds to an increase in Reynolds number of 0.00168 to 35.930, which does not indicate turbulent behavior. However even at lower Reynolds numbers, the increase in flowrate could still lead to higher shear and impact stresses by that an increase in droplet reduction (Gothsch et al., 2011; Katritsis et al., 2007). The usage of all three membranes (Ni-based superalloy membrane, sintered disc and perforated steel disc) shows the smallest droplets of all setups in the range of a nanoemulsion. Usage of the nickel-based superalloy membrane together with the perforated steel disc alone led to similar droplet size distributions than the membrane and both discs together. However, in the experiment, the superalloy membrane was pressed into the large drilled pores of the steel discs and was irreversibly damaged. This can explain the shoulder of the droplet size distribution at the size of 2–3 μm. The results are consistent with the classical assumption that smaller pores also result in smaller droplet sizes (Nagasawa et al., 2020). Given these results, the conclusion that the support discs show no significant influence on the droplet size, but are needed for stabilizing the superalloy membrane, can be drawn.

### 3.2 Effect of the type of membrane structure

In order to analyze the influence of the membrane structure on the premix emulsification process, three different membranes were used. Two of them were superalloy membranes with different structures of interconnected pores and one was a PE membrane with straight through pores.

**Fig. 5:**
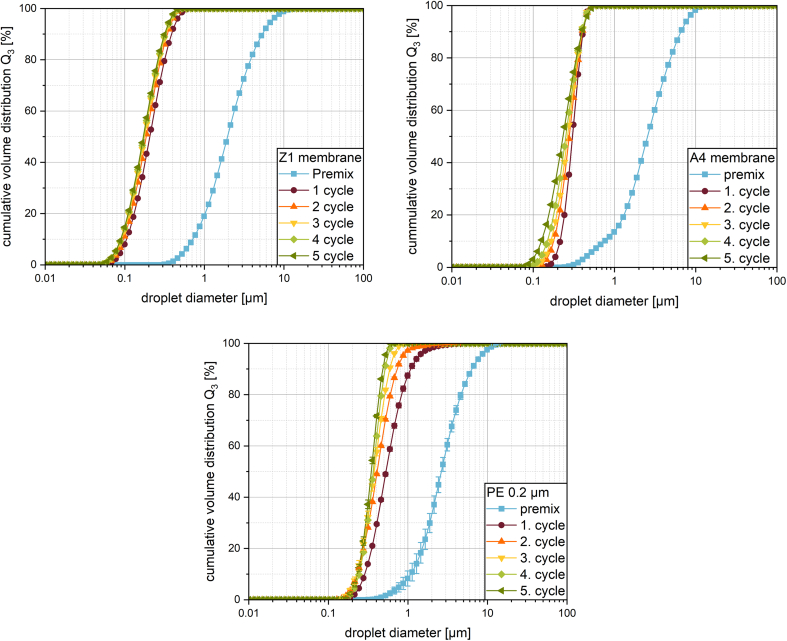
Droplet size distribution for increasing the number of emulsification cycles with metal membranes with A4 (top left) and Z1 membrane structure (top right) as well as a track etched polyethylene membrane with 200 nm pores (bottom) at 120 bar.

For the Z1 membrane structure with pore sizes of 143 ± 105 nm and a narrow pore size distribution, droplets with a median size of about 206 nm were achieved after one emulsification cycle (see Fig. 5, top left). This is a more than tenfold reduction in mean droplet size from a broader droplet size distribution to a monomodal one compared with the premix. With a span of about 1.26, a stable, colloidal, and monodisperse emulsion was achieved. With further emulsification cycles, only little additional droplet breakup was detected. Five emulsification cycles resulted in a median droplet size of around 171 nm with a span of approximately 1.17. The droplet size distribution retained a similar shape, but was shifted slightly towards smaller droplet sizes (see Fig. 5, top left, and Table 3).

**Table 3:** Median droplet size, span and droplet to hydraulic diameter ratio after emulsification at 120 bar using different membranes for an increasing number of emulsification cycles, with 0 being the premix.

Membrane ID	Pore width [nm]	Hydraulic diameter [nm]	Cycle [−]	Median droplet size [nm]	Span [−]	Droplet to hydraulic diameter ratio [−]
Z1	143 ± 105	192 ± 295	0 1 2 3 4 5	1950 ± 26 206 ± 1 186 ± 1 177 ± 1 173 ± 1 171 ± 1	2.318 ± 33 1.258 ± 1 1.297 ± 1 1.323 ± 1 1.338 ± 4 1.344 ± 1	- 1.073 0.969 0.922 0.901 0.890
A4	342 ± 142	469 ± 225	0 1 2 3 4 5	2553 ± 21 305 ± 1 278 ± 1 266 ± 1 253 ± 1 233 ± 1	2.281 ± 20 0.648 ± 2 0.808 ± 2 0.877 ± 4 0.979 ± 4 1.167 ± 1	- 0.650 0.593 0.567 0.539 0.497
PE	200	200	0 1 2 3 4 5	2627 ± 119 531 ± 5 410 ± 2 376 ± 7 365 ± 1 345 ± 3	2.015 ± 115 1.494 ± 67 1.231 ± 13 0.965 ± 29 0.744 ± 2 0.732 ± 18	- 2.655 2.050 1.880 1.825 1.725

In comparison, the A4 membrane structure, with a pore size of 342 ± 142 nm, did not reach a droplet size as small as the Z1 structure in one cycle (see Fig. 5, top right), yielding about 305 nm median size with a span of approximately 0.65. The size reduction in comparison to the premix is still eightfold. Unlike the Z1 structure, the A4 structure showed a stronger droplet breakup with increasing number emulsification cycles. It should be noted, that this decrease in median droplet size went along with an increase in the span of the droplet size distribution. The minimal droplet size was reduced while the maximum droplet size stayed similar to the results of the previous emulsification cycles, broadening the distribution width (see Fig. 5, top right, and Table 3).

In comparison with the two new types of membranes, the PE membrane with a pore width of 200 nm (according to the manufacturer) resulted in droplets with a median size of 531 nm after the first cycle. This equates a fivefold reduction in droplet size in comparison to the premix. With an increase in cycles, the median droplet size is reduced and the span is reduced (see Fig. 5, bottom, and Table 3). This reduction in droplet size over multiple cycles corresponds to typical results for multi-cycle emulsification in literature (Surh et al., 2008; Vladisavljević et al., 2004).

These differences in membrane behavior can possibly be explained by their structure. The larger droplets produced by the A4 membrane correlate with the larger pore size of 342 ± 142 nm in this structure. The smaller pore size of the Z1 membrane of 143 ± 105 nm enables a production of smaller droplets given the same formulation and process parameters (Nagasawa et al., 2020). As already reported for the production of agarose beads with SPG- and polyethylene-membranes (Zhou et al., 2009) and O/W- and W/O/W -emulsions with SPG-membranes (Umeda et al., 2025; Vladisavljević et al., 2006), the droplet size depends strongly on the pore size. Accordingly, the narrower droplet size distribution after one cycle using the A4 membrane is a surprising result given the wider pore size distribution of this membrane. An explanation for this could be the number of active pores of this membrane. It was already shown, that the number of active pores increases with pressure (van der Zwan et al., 2006) and the number of active pores in direct membrane emulsification using SPG membranes is low compared to the total membrane area (Vladisavljevic et al., 2007). Active in this case means, that these pores are transporting the disperse phase, i.e. oil droplets through the membrane and not only the continuous phase. The process of pressing a disperse system, like an emulsion, through a membrane can be compared to a filtration process. When not enough energy for the deformation or breakup of oil droplets, being typically larger in size than the pores, is introduced into the system, the droplets will not be able to pass through the membrane. In that case, it can be expected that the energy will be used for pressing the continuous phase through the membrane, as is the principle of a filtration process. Thus, it can be assumed that the larger pores of the A4 membrane, offering the path of least resistance for the droplets due to the higher mass flow of the continuous phase and reduced droplet deformation, were responsible for conveying the majority of the oil. While each emulsification cycle in this experiment was performed using the same pressure of 120 bar, the characteristics of the emulsion changed drastically after the first cycle, given the fact that there were no more droplets with a size larger than 1 μm present. Thus, for the second emulsification cycle with the A4 membrane, it appears that the droplets were transported through a wider range of pore sizes. While some were transported through the same large pores as before some were able to be deformed enough to pass through the smaller pores of the wide pore size distribution. This resulted in further partial breakup of the droplets resulting in a broadening of the droplet size distribution.

The phenomenon of increase in span with cycles was not seen as drastically for the Z1 membrane. With this membrane, the very small droplets could already be produced after one cycle, possibly due to the narrower and finer pore size distribution in this membrane. However, the span was roughly double that of the A4 membrane. Further emulsification cycles reduced the droplet sizes slightly and did not result in a broadening of the droplet size distribution. This can probably be explained via the pore size distribution: The significantly smaller droplets in the second cycle were not being pressed through smaller pores as with the A4 membrane, because there were no pores present that showed a significant difference in size.

As the pore structure of the PE membrane is fundamentally different to that of the superalloy membranes, the droplet is pressed through a narrow tube almost without any tortuosity. Therefore, the droplet breakup will be different. The first cycle with the PE membrane resulted in the highest droplet to hydraulic diameter ratio of all membranes used (see Table 3). This raises the hypothesis that a tortuous membrane causes a stronger droplet breakup than in a straight through membrane. However, the straight through pore structure was able to reduce the median droplet size with further cycles while reducing the span at the same time. In contrast to the metal membrane, the droplet size distribution of the PE membrane showed a reduction in the maximum droplet size, while the minimum droplet size showed constant values. This indicates that the droplet breakup mechanisms acting in the very homogeneous PE membrane have a possible minimal droplet size that can be produced. When considering this, one has to keep in mind, that the viscosity ratio between disperse and continuous phase was 15.3 (viscosity disperse phase 23.86 mPa s and continuous phase 1.56 mP s). Grace et al. reported that no droplet breakup was reachable with a viscosity ratio above 4 with only shear flow in laminar conditions in an unconfined shear field (Grace, 1982).

At the pressures used here, there is a possibility that the PE membrane may be temporarily deformed mechanically during the test. Visually, the membrane showed no cracks or other damage after the test. Since a smaller and narrower distribution was already observed after one cycle than when using only the support structures in section 3.1, it can be concluded that the structure of the membrane was not negatively affected to such an extent that the values are not comparable.

In any case, it can be reported that a monomodal emulsion with a median droplet size of around 300 nm for the A4 and around 200 nm for the Z1 membrane can reliably be produced with one emulsification cycle.

### 3.3 Influence of pore length on emulsification

**Fig. 6:**
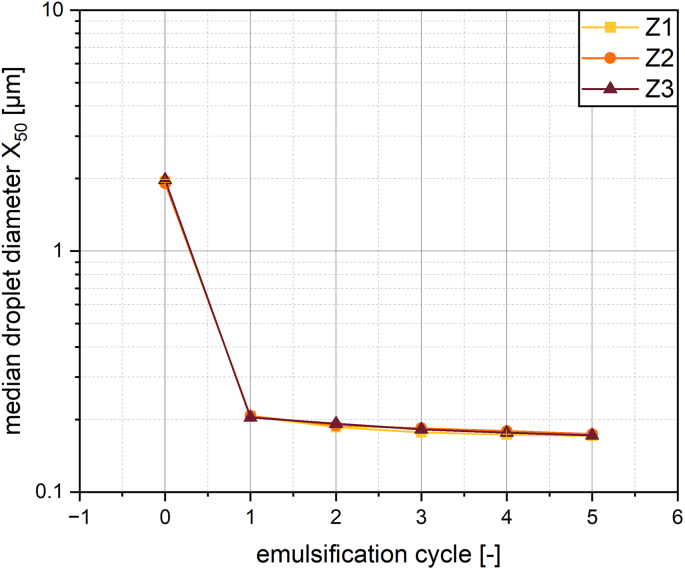
Median droplet size for increasing the number of emulsification cycles with superalloy membranes of similar membrane structure but different production parameters at 120 bar.

The results in chapter 3.2 show a difference in emulsification behavior for the two different membrane structures. To investigate if a difference in droplet size is to be expected from membranes with a similar structure but different production parameters the three membranes Z1, Z2 and Z3 were used for emulsification under the same parameters as before. The membranes were treated at the same temperature and stress by different treatment times to different elongations, which resulted in similar pore width, but different pore length (see Table 1). It is important to note that the pore length referred to here does not mean the thickness of the membrane, but the dimension orthogonal to the pore width in the top view of the membrane, while the pore width represents the smallest dimension of the pore. Keeping the pore width constant, the droplet sizes after emulsification were very similar for the different membranes. All three membranes showed the most drastic droplet breakup in the first cycle and only little breakup with a further increase in cycles (see Fig. 6). Therefore the pore width and not the pore length is decisive for the droplet size.

### 3.4 Effect of emulsification pressures

**Fig. 7:**
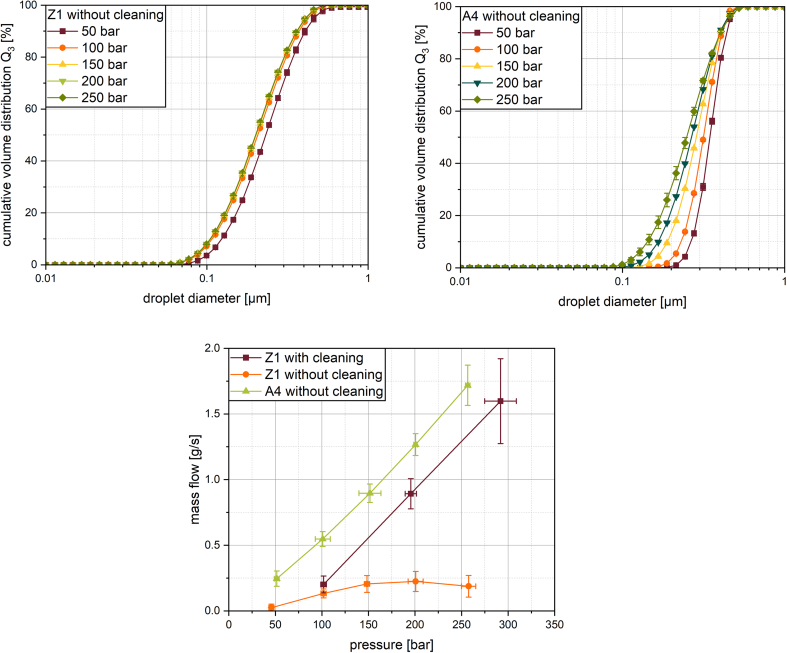
Droplet size distribution after one cycle of premix membrane emulsification with Z1 (top left) or A4 (top right) membrane structure at different pressures (50–250 bar) and the associated mass flows (bottom).

Given the nature of the membranes, a higher flow resistance against pressure is expected for metal membranes compared to polymer membranes. Due to the previous results showing only little further breakup with increased cycles for the metal membranes, only one cycle was performed in the following experiment. The emulsification was performed using the same membranes as before (Z1 and A4). The first experiment was performed with increasing pressures directly one after the other, without a cleaning procedure in between. For this first experiment, the mass flow through the membrane increased linear with an increase in pressure for the A4 membrane (see Fig. 7). For the Z1 membrane in contrast, the mass flow stagnated at a pressure increase from 150 to 200 bar and even decreased at a pressure of 250 bar (see Fig. 7 at the bottom), which strongly hints at fouling (Tummons et al., 2020). The smaller and more uniform pores of Z1 (see Fig. 2, Fig. 3) provide a greater resistance when pressing an emulsion through the membrane, resulting in a lower mass flow. Additionally, it is also reasonably more prone to fouling than the larger pores in the A4 membrane due to the pore size and shape with the narrow opening that in sum causes higher specific surface areas. The slower flow through the membrane additionally allows deposits to settle more easily compared to higher flow rates that may remove deposits mechanically. It is reasonable to assume similar material parameters for both of the membranes regarding the polarity and interfacial energy. Given the smaller pore size in the Z1 membrane, this is a common problem in filtration of oily wastewaters, where smaller pore sizes often are associated with fouling (Nagasawa et al., 2020).

The results regarding the droplet sizes were similar to those regarding increased emulsification cycles. For the A4 structure, an increase in pressure again lead to a decrease in the minimum droplet size and a broadening of the droplet size distributions (see Fig. 7 (top right)). Once again, the maximum droplet size remained similar, while the minimal droplet size was reduced. The exact cause of this phenomenon is not mechanistically proven, yet. However, the increase in mass flow may provide one hypothesis: One of the mechanisms, by which droplets are broken up, is by elongation flow at the entry of the membrane (van der Zwan et al., 2006) or by breakup of an elongated filament of oil into smaller droplets (Khakhar and Ottino, 1987). When a droplet experiences an elongational stress field, it deforms into a long rod while initially remaining intact as a continuous droplet. However, elongated oil droplets have a higher probability of breaking up into small droplets due to Rayleigh-Taylor instabilities. An increase in breakup with increasing flow through a constriction was already shown by Rosenfeld et al. (Rosenfeld et al., 2014). Thus, an increase in pressure leads to an increase in flow velocities and consequently in droplet filaments ripping apart, but this cannot be the single mechanism responsible for the increase in breakup, because it did not show up as pronounced for the Z1 membrane.

A second hypothesis connects to the already mentioned activity of pores. Due to an increased energy input in form of higher pressure into the system, oil droplets may also be captured by flow into and pressed through smaller pores in the membrane. As discussed and shown before, a narrower pore equals smaller droplets at the end of the process (Nazir et al., 2011). Given the broader pore size distribution of the A4 membrane, the result is a wider droplet size distribution, again.

The findings, which show that the mass flow can be increased with rising pressure and that particularly comparable droplet sizes were generated with the Z1 membrane, highlight the potential of these metal membranes. Despite the need for cleaning, the membrane proves to be reusable. From a business perspective, this demonstrates the potential of using the membranes to reduce running costs during operation and provide a more sustainable footprint. However, it should be noted that significantly higher pressures are used in this process than in other premix membrane emulsification processes. This requires more powerful pumps for supply and pipes for transport. This may inevitably increase capital expenditure.

### 3.5 Effect of fouling and cleaning

**Fig. 8:**
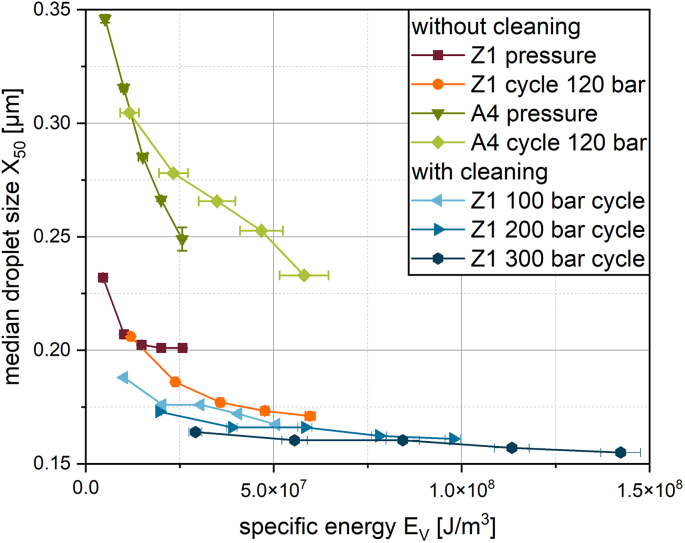
Mean droplet size (X_50_) as a function of the specific energy input varied by the number of cycles or the process pressure in one cycle.

In order to reduce the impact of fouling on the resulting mass flow, the experiment was repeated with a second Z1 membrane. Five consecutive cycles were performed for one constant pressure without a cleaning regime in between cycles. After the experiment was performed, the membrane was cleaned according to the specifications given in chapter 2.2.2. This procedure should ensure that the membrane can be used unimpaired for the tests with the other pressures. With this procedure, the mass flow showed a linear increase with pressure for Z1 as well (the mass flow for the first cycle for each pressure is shown in Fig. 7 (bottom). Even though the mass flow increased significantly, only a small reduction in median droplet size was found (see Fig. 8). A second Z1 membrane with the same manufacturing conditions was used for this experiment. Presumably due to production-related deviations, slightly smaller droplet sizes were achieved with this membrane. The median droplet size was 188 ± 1 nm after one emulsification cycle compared to 207 ± 1 nm in the experiments without cleaning (see Fig. 5 (top left)). However, the trend in droplet size distribution remained the same. An increase in pressure, which in this case was accompanied by a significant increase in mass flow, led to a slight shift in the overall distribution towards smaller droplet sizes. No broadening of the distribution towards smaller droplet sizes was observed (see Fig. S1 and S2 in the supplementary material). The increase in the number of cycles again showed only slight further droplet break-up in the Z1 membrane as found before (see Fig. 5 (top left)).

Due to the high difference in pressures and resulting mass flows, a wide range of flow conditions with corresponding Reynolds numbers are covered (see Table 4). However, Re are fairly low with the highest value being 0.0046 for the A4 membrane with a mass flow of 1.72 ± 0.15 g/s and thus significantly lower than the values reported in chapter 3.1 with the highest Re at 35.930. As stated before, it can be concluded, that no turbulence following the superalloy membrane was responsible for the droplet breakup. However, since in this case the emulsification was carried out with all support structures and a membrane, the mass flow was considerably lower, which also resulted in a lower Re. The Z1 membrane shows almost the same droplet sizes with and without cleaning, i.e. at very different flow rates (see Table 4 and Fig. 8). One possible explanation for this is based on the reduction of the free flow area, which results in a constant flow velocity in the remaining open pores. However, this cannot be proven on the basis of the presented data. It is possible that there is a critical pore size for fouling in this process that lies between the pore sizes of membranes A4 and Z1, so that stronger fouling is observed at Z1.

**Table 4:** Flow parameters for emulsification for one cycle with different pressures for consecutive and emulsification with cleaning between cycles.

Membrane ID	Pore width [nm]	Hydraulic diameter [nm]	Pressure [bar]	Mass flow [g/s]	Reynolds number [−]	cce [nm]
Z1 (without cleaning)	143 ± 105	192 ± 295	45.73 ± 2.69 101.76 ± 2.87 148.40 ± 2.90 200.67 ± 8.10 257.57 ± 7.63	0.025 ± 0.027 0.133 ± 0.034 0.205 ± 0.064 0.225 ± 0.077 0.188 ± 0.083	0.00004 0.00020 0.00031 0.00034 0.00028	61.20 95.25 97.97 88.14 71.13
Z1 (with cleaning)	143 ± 105	192 ± 295	101.78 ± 3.02 195.47 ± 6.12 291.81 ± 16.95	0.202 ± 0.064 0.892 ± 0.115 1.598 ± 0.323	0.00030 0.00134 0.00240	117.27 178.04 194.96
A4	342 ± 142	469 ± 225	51.46 ± 2.63 100.87 ± 8.09 151.60 ± 11.81 200.52 ± 1.87 256.63 ± 2.36	0.245 ± 0.058 0.547 ± 0.057 0.896 ± 0.070 1.267 ± 0.083 1.718 ± 0.153	0.00090 0.00201 0.00329 0.00465 0.00631	181.96 194.04 202.59 209.41 215.61

Using eqs. (5), (6), under the given conditions of the membrane pores a simplified assessment can be made as to whether, further flow can occur if the membrane is wetted with oil. To achieve the observed mass flows with a liquid phase made entirely up of oil, the applied pressure according to the calculations needs to be higher than 63000 bar. Alternatively, with the applied pressure and a membrane completely wetted with oil, a maximum mass flow of 0.04 g/s would have been possible for membrane A4. This corresponds to approximately 0.035 % of the observed flow rate. For this reason, it is to be assumed that while some local wetting might occur, the membrane is not completely wetted with oil and that the resulting flow is still made up of the premix emulsion. However, this also indicates that if oil wetting of the membrane occurs, the applied pressure is insufficient to flush the pores free again.

Another parameter to compare for the membranes is the cylindrical capillary equivalent pore diameter (cce), which gives a pore diameter if the membrane consisted of straight through pores. The low mass flow in the experiment with the consecutive pressure increase without cleaning for the Z1 membrane results in a fairly low cce compared to the pore width measured via SEM. The experiment including a cleaning regime between the tests results in pore sizes similar to those measured by SEM. Additionally, the cce rises with increasing pressure. This indicates that the increase in mass flow is greater than physically expected for the membrane. The cce results for the A4 membrane underestimate the pore width measured by SEM. The cce again rises with an increase in pressure, but even the highest value of 215.61 nm is lower than the measured pore width of 342 ± 142 nm and even more so with the hydraulic diameter of 469 ± 225 nm. The authors do not assume that an increase in pressure leads to a physical expansion of the pores. This is partly because the material used has a high mechanical strength and partly because no change in the pore structure was observed on SEM images after emulsification. Rather, these results support the theory that an increase in pressure is accompanied by an increase in active pores. The increase in cce could also be related to the change in the overall viscosity of the emulsion due to the change in droplet size of the dispersed phase. A stronger droplet breakup could influence the viscosity in such a way that the cce increases. However, the size of the droplets is within the minimum pore dimensions, so that the bulk properties of the emulsion can probably not be argued here, but individual droplet properties are decisive.

The experiments of the Z1 membrane are in contrast to the previous reports on Ni-based superalloy membranes by Kohnke et al. (Kohnke et al., 2018), who reported little to no fouling for these membranes. However, a membrane with a pore structure similar to that of A4 was also used in that publication and this membrane also showed very little to no fouling in the tests shown here. The fact that the membrane structure Z1 nevertheless shows fouling can presumably be attributed to the smaller pores already mentioned above and the associated greater tendency to foul.

The difference in surface properties of different Nickel compounds need to be taken into account with regard to fouling. Nickel components or nickel coatings are frequently used in research into water treatment and the oil and water separation. In these applications they proved to reduce fouling and enable the retention of a majority original mass flows (Tan et al., 2024; Wei et al., 2024). Accordingly, those Nickel compounds possess different superficial interaction profiles than the surfaces of our Nickel-based super alloy membranes, which do show fouling. Future research may focus on the functionalization of the membrane surfaces with comparable Nickel-based anti-fouling coatings.

### 3.6 Influence of specific energy input

The results can also be plotted and compared using the specific energy input.

When pressing the premix through the membrane, the setup used for these experiments allowed for a constant pressure. Using this, the specific energy input can be approximated via eq. (2). For the A4 membrane, the increase in pressure and in cycle number resulted both in an approximately linear decrease in mean droplet size (see Fig. 8), but with a different size reduction rate.

For the increase in cycle number and pressure, the mean droplet sizes for Z1 do not show a linear decrease, but appear to converge to a limit value in both experiments performed without a cleaning regime in-between emulsifications. The decrease decelerates after 10^7^ J/m^3^ (corresponding to one cycle of 100 bar). With a cleaning regime between emulsifications, the median droplet size continues to decrease for an increase in pressure (see Fig. 8). This can be concluded from the decrease in droplet size of the first cycles of the different pressures. The resulting decrease in droplet size is not as drastic as for the A4 membrane, but smaller droplet sizes were obtained. The increase in cycle number for the different pressures again appears to converge to a limit value.

When consulting the specific energy used for both processes, it appears that the increase in pressure is energetically more efficient than an increase in cycles. Thus, the stress intensity is more crucial than the stress frequency applied for emulsification with superalloy membranes. Accordingly, the energy input required to meet a certain mean droplet size by increasing the pressure is lower than increasing the cycle number at lower pressure.

### 3.7 Effect of membrane thicknesses

In order to investigate the influence of the membrane thickness, the Z2 and A4 membrane were studied in three different thicknesses of 0.1, 0.2 and 0.3 mm, respectively.

**Fig. 9:**
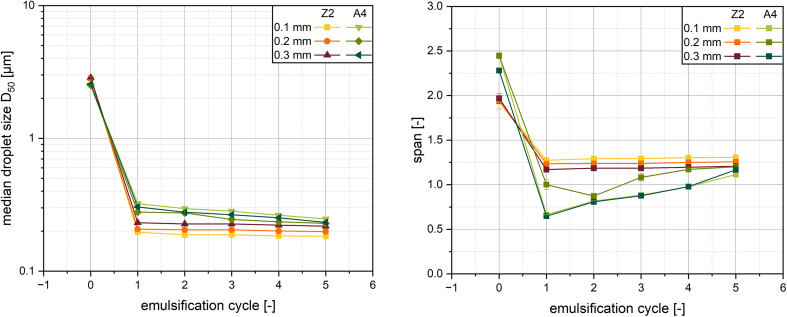
Mean droplet size X_50_ (left) and span (right) after multiple membrane emulsification cycles at 120 bar.

Varying the thickness of the superalloy membranes caused little to no change in emulsification results (see Fig. 9 and Fig. S3 in the supplementary material). The resulting droplet size distributions when using the same process and formulation parameters for both membranes were similar to those reported in chapter 3.3. An increase in cycles again resulted in a slight shift of the whole distribution towards smaller droplets for the Z2 membrane and a broadening of the distribution towards smaller droplets for the A4 membrane. For the A4 membrane, no clear ranking of the results became apparent. Given the tortuous inner structure of the alloy membranes, it can be assumed that an increase in thickness of the membrane comes with an increased number of stress events for one droplet when passing through. However, these stress events do not seem to result in a reduced droplet size with the parameters used. This indicates that for this combination of process- and formulation parameters, localized shear forces inside the membrane are not the significant mechanisms of droplet breakup. Since droplet entry and exit have to occur in any case, regardless of the membrane thickness, it can be argued that the elongational flows on the membrane inlet is responsible for the main droplet breakup, but they will probably not be solely responsible. This theory suggests that during droplet breakup in this membrane, the droplet is deformed into a long filament by the elongational flows at the inlet and then broken up by shear in the membrane. Since, according to this hypothesis, the breakup of the droplets occurs very early after the droplet enters the membrane, a lack of influence from an increase in membrane thickness can be explained. The shear stress in the 0.1 mm thick membrane was enough to breakup droplets which pass through the membrane over a length of at least 300 times the pore width, not taking tortuosity into account.

The results for the Z2 membrane are also very similar regardless of membrane thickness but they are ordered with the thinnest membrane resulting in the smallest droplets. This might again indicate that not the number of stress events inside one membrane is the most important part of premix emulsification using this type of superalloy membrane, because the first shear events after the initial droplet elongation breakup the droplet. Further shearing of the droplet is not sufficient to result in further breakup, and in theory could even lead to droplet coagulation and increase in median size. However, this cannot be concluded from the available data. The median droplet size also affects the span with the thinnest membrane resulting in the broadest distribution. This result only partly aligns with results previously reported for the production of agarose beads with polyethylene membranes of varying thicknesses via premix membrane emulsification. An increase in membrane thickness from 1 to 2 mm resulted in significant reduction in bead size with a more uniform distribution (Zhou et al., 2009).

The thinnest membrane in this experiment was still 300 to 500 times thicker than the average droplet diameter. This may mean that a possibly existing, critical minimal membrane thickness, at which the shear would not be sufficient for droplet breakup after elongation, was not reached. This may possibly lie in the order of magnitude of the pore widths. Such a low thickness can probably not be studied in reality due to the minimal membrane thickness being technically limited by its production process and mechanical properties.

### 3.8 Effect of emulsifier concentrations

**Fig. 10:**
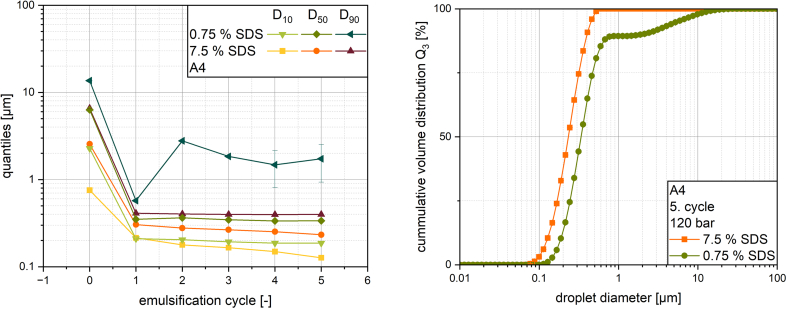
Droplet size quantiles (X_10_, X_50_ and X_90_) and droplet size distribution after five membrane emulsification cycles at 120 bar with 0.75 and 7.5 % SDS.

When varying the formulation with different concentrations of SDS, the droplet size distribution broadened with a reduction of the emulsifier content with the A4 membrane (Fig. 10). With both concentrations, similar minimal droplet sizes were achieved after one emulsification cycle. For the lower concentration, the distribution was widened towards larger droplet sizes due to increased X_50_ and X_90_. Especially after the second cycle, an increase in larger droplets can be detected. Due to identical process parameters, the mechanisms of droplet breakup are assumed to be similar in both experiments. Therefore, the difference in achieved droplet size can be attributed to the influence of the emulsifier on droplet stabilization or coalescence. A more detailed explanation may be provided by DeRoussel's model of overemulsification (DeRoussel et al., 2001). The droplet sizes achieved with the lower emulsifier concentration are in accordance with the overemulsification theory, in which a final steady state is reached after running through an initial minimum. According to this model, overemulsification occurs, when the minimum droplet size that can be deformed by a flow is greater than the maximum droplet size that will coalesce in a flow. Applied to the experiment conducted here, it can be concluded that in the first cycle, the initial droplet sizes were above the minimum deformable size. Consequently, the droplets were deformed, broken up, and smaller droplets were produced. If these are now subjected to further stress through another cycle, they are unable to be deformed further, instead the flow causes the droplets to coalesce as the maximum coalescible droplet size was higher than the given size. This was only the case for the lower emulsifier concentration, as the droplet sizes relevant here depend on the shear rate, the viscosity of the continuous and dispersed phases, and the interfacial tension. The reduction in emulsifier corresponds with an increase in interfacial tension, which changes the minimum deformable size and maximum coalescible size and subsequently promotes overemulsification. A higher concentration of emulsifier results in a larger amount of free emulsifier in the solution, which can migrate to the interface of newly formed droplets to stabilize them. When assuming that one SDS molecule covers roughly 55 Å^2^ on the surface of an oil droplet (Llamas et al., 2018) even the lower concentration of 0.75 % SDS should be able to cover the whole surface of the oil droplets assuming a median size of 346 nm with still around 78 % SDS being free in solution. And even when calculating with the X_10_ of 210 nm roughly 64 % SDS should be free in solution. Nevertheless, given emulsification is not an ideal process, the presence in the right location and the speed at which the emulsifier migrates to the interface have a major influence on the result. As emulsification through the input of mechanical energy is an interplay between droplet breakup and coalescence, it can be assumed, that more newly formed droplets coalesced due to slower stabilization at a lower emulsifier concentration. Further tests with varying emulsifier concentrations and the use of other emulsifiers are necessary to prove this hypothesis with additional results. The influence of different stabilization mechanisms and emulsifier migration rates could be particularly useful here to deepen the understanding of droplet breakup and coalescence in this process in future research.

## 4 Conclusion

Ni-based superalloy membranes proved effective for premix membrane emulsification to achieve sufficiently small droplet sizes for pharmaceutical application with droplet sizes less than or equal to 200–450 nm in a single cycle. Two different nanoporous structures were tested. The membrane with narrower, elongated pores showed little further droplet breakup with increasing pressure or cycle number. In contrast, increasing either cycle number or pressure using the wider and more isotropic pored membrane structure resulted in decreased mean droplet sizes but an increased distribution width. The cause for such different results may be found in the difference in pore geometry and the changed droplet-specific flow patterns. Broader pore size distributions favor flow through larger pores for droplet breakup at low energy inputs, with smaller pores contributing under higher pressure and thus widening the droplet distribution. This is unlike in straight-through polymer membranes, which can reduce droplet sizes and narrow droplet size distributions with an increase in cycles. However, such membranes do not achieve the small droplet sizes and high efficiency with one emulsification cycle as the metal membranes do.

The findings point to elongational flow when entering the pores as the dominant breakup mechanism. There droplets are elongated into filaments and disintegrate either due to prolonged stretching or shear imposed by the membrane's tortuosity. Promising for the industrial application is that structured metal membranes provide robust emulsification processes with low effect of pressure fluctuations and cycle numbers.

Future works will study the influence of the emulsifier regarding emulsifier-membrane interactions, kinetic parameters (emulsifier migration rates) and static parameters (interfacial tensions and wettability of the membrane). Furthermore, formulation effects will be deeper studied by applying emulsifiers with higher relevance for parenteral use, elucidating the extent, to which the results of the SDS are transferable.

## CRediT authorship contribution statement

**Daniel Jupke:** Writing – original draft, Methodology, Investigation, Conceptualization. **Janik Marius Lück:** Writing – original draft, Resources. **Joachim Rösler:** Writing – review & editing. **Jan Henrik Finke:** Writing – review & editing, Methodology, Conceptualization. **Arno Kwade:** Writing – review & editing.

## Declaration of competing interest

The authors declare the following financial interests/personal relationships which may be considered as potential competing interests:

Daniel Jupke reports financial support was provided by German Research Foundation. Daniel Jupke reports article publishing charges was provided by Project DEAL. Janik Marius Lueck reports financial support was provided by German Research Foundation. Joachim Roesler reports financial support was provided by German Research Foundation. Jan Henrik Finke reports financial support was provided by German Research Foundation. Arno Kwade reports was provided by German Research Foundation. If there are other authors, they declare that they have no known competing financial interests or personal relationships that could have appeared to influence the work reported in this paper.

## Data Availability

Data will be made available on request.

## Appendix A Supplementary data

Supplementary material Image 1
