# Supplementary material for: Exploring the influence of the nanoporous structure of nickel-based superalloy membranes on emulsification performance
Source: Int J Pharm X. 2025 Jul 27;10:100369. doi: 10.1016/j.ijpx.2025.100369 (PMC12336008; doi:10.1016/j.ijpx.2025.100369)
Supplement: Supplementary file 1 — Supplementary material [file mmc1.pdf]

Supplementary Material

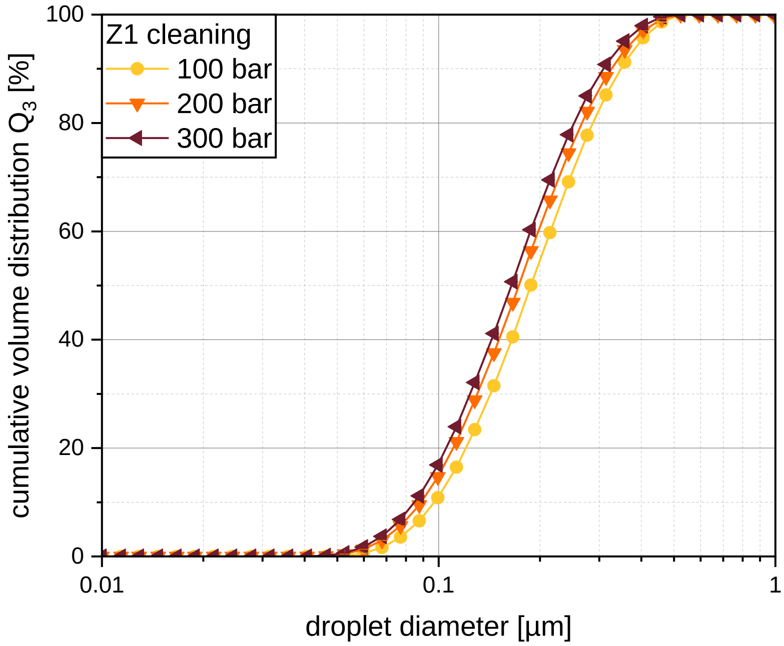

Figure S1: Droplet size distribution after one cycle of premix membrane emulsification with membrane Z1 with cleaning in between emulsifications

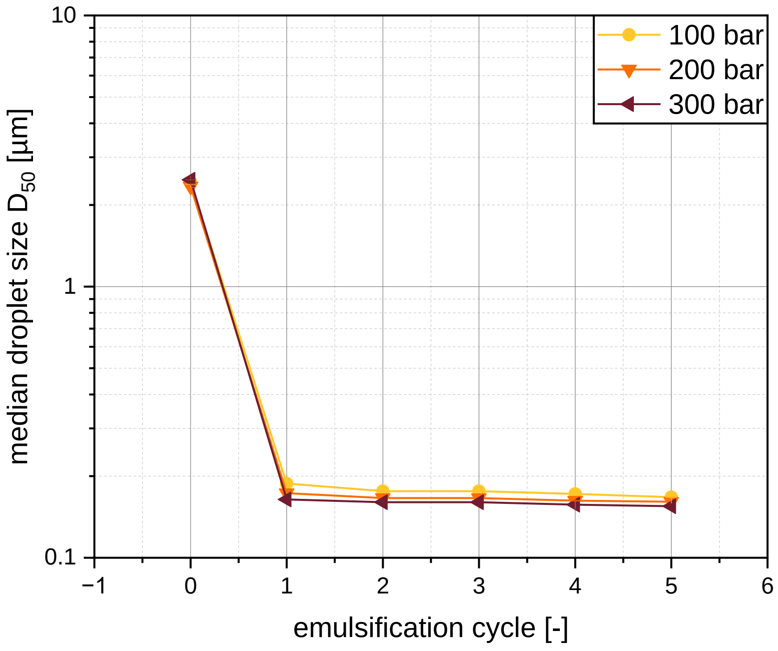

Figure S2: Mean droplet size X_{50} after one to five consecutive cycles of premix membrane emulsification with membrane Z1

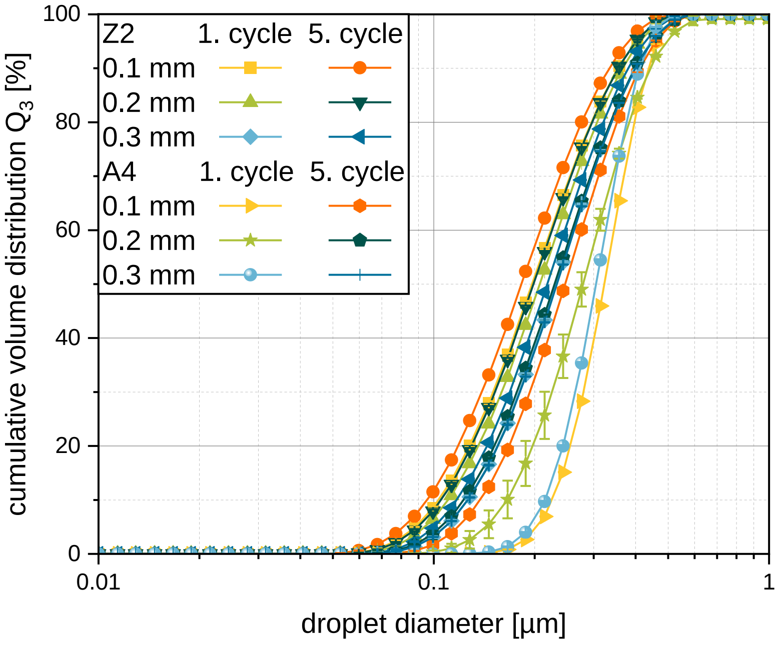

Figure S3: Droplet size distribution after one and five cycles of premix membrane emulsification with membranes Z2 and A4 of varying thickness
